# Supplementation with Branched-Chain Amino Acids Induces Unexpected Deleterious Effects on Astrocyte Survival and Intracellular Metabolism with or without Hyperammonemia: A Preliminary In Vitro Study

**DOI:** 10.1155/2021/7615126

**Published:** 2021-09-10

**Authors:** Ting Wang, Kazuyuki Suzuki, Toshimi Chiba, Keisuke Kakisaka, Yasuhiro Takikawa

**Affiliations:** ^1^Division of Hepatology, Department of Internal Medicine, Iwate Medical University, Morioka, Iwate, Japan; ^2^Division of Internal Medicine, Department of Oral Medicine, Iwate Medical University, Morioka, Iwate, Japan

## Abstract

**Introduction:**

Ammonia is a key component in the pathogenesis of hepatic encephalopathy. Branched-chain amino acids (BCAA) have been reported to improve the symptoms of HE induced by hyperammonemia; however, we recently reported that ammonia increases intracellular levels of BCAA and exerts toxic effects on astrocytes.

**Objectives:**

This follow-up study was designed to confirm the direct effects of BCAA on human astrocytes and clarify their underlying mechanisms using metabolome analysis and evaluation of associated signaling.

**Methods:**

We performed cytotoxicity and cell proliferation tests on astrocytes following BCAA treatment with and without ammonium chloride (NH_4_Cl) and then compared the results with the effects of BCAA on hepatocytes and neurons. Subsequently, we used metabolomic analysis to investigate intracellular metabolite levels in astrocytes with and without BCAA treatment.

**Results:**

The astrocytes showed increased leakage of intracellular lactate dehydrogenase and reduced proliferation rate upon BCAA treatment. Interestingly, our analysis showed a BCAA-induced impairment of intracellular glycolysis/glyconeogenesis as well as amino acid and butyric acid metabolism. Furthermore, BCAA treatment was found to cause decreased levels of Glut-1 and phosphorylated GSK-3*β* and mTOR in astrocytes.

**Conclusions:**

Although further investigations of the effect of BCAA on human astrocytes with hyperammonemia are needed, our work demonstrates that BCAA supplementation has direct negative effects on astrocyte survival and intracellular metabolism.

## 1. Introduction

Plasma and brain amino acid (AA) imbalance, particularly low concentration of branched-chain amino acids (BCAA) and high concentration of aromatic amino acids (AAA) in plasma, closely participates in the pathogenesis of hepatic encephalopathy (HE) in liver cirrhosis (LC) and/or portal-systemic shunt, resulting in the disturbance of neurotransmission in the brain [1]. In general, orally administered BCAA is a clinically used nutritional therapy to alleviate protein-energy malnutrition (PEM) in patients with LC [2, 3]. Intravenous BCAA-enriched amino acid solution (Fischer's solution) is usually used in the initial stage of overt HE, based on the false neurotransmitter theory [4–6]. Conversely, high AAA concentration in plasma induces neurological disturbances through the increased synthesis and release of false neurotransmitters in the brain, derived from AAA. BCAA supplementation can improve neurological disturbances by competitively reducing AAA concentrations in the brain via the blood-brain barrier (BBB).

Astrocytes play important roles in central nervous system (CNS) function and in maintaining the BBB through their “end feet,” which interact with the structural components of the barrier. Recent studies have found that astrocytes determine both the functional and structural architecture of the adult brain [7]. Astrocyte structural abnormalities and dysfunction may lead to disruption of the BBB [8]. Beyond that, astrocyte failure is implicated in the pathogenesis of neurological disorders such as astrocyte swelling in Alzheimer type 2 cells that are reported during the initiation of HE [9].

Ammonia is a key substance in the pathogenesis of HE [10, 11]. We recently reported that ammonia increases the intracellular levels of BCAA and of their neurotoxic derivatives, such as 3-methyl-2-oxovaleric acid in human astrocytes. Our data suggest that BCAA can be toxicants and facilitate the cytotoxic activity of ammonia on astrocytes [12].

Previous research has highlighted the importance of evaluating the effects of BCAA overdose on the brain to determine BCAA threshold values for adverse effects [13]. However, no toxic BCAA accumulation has been detected in the brain except for that in the Maple Urine Symptom Disease (MUSD), a very rare genetic defect, which could not serve as a suitable model for BCAA overdose in healthy individuals [14]. Additionally, we previously determined the BCAA concentration in the peripheral blood, cerebrospinal fluid (CSF), and brain tissues of dogs with hyperammonemia as well as Eck fistula (a model of portal-systemic shunt); we found that the BCAA concentration in the blood was significantly lower than that in the control, while the BCAA concentration in CSF did not decrease significantly [15]. These findings have also been reported by other laboratories [16, 17]. Furthermore, BCAA concentration in brain tissue showed similar levels to the control [15]. These results suggest that BCAA levels may not decrease in the CSF and brain tissue of HE patients and that additional BCAA supplementation may increase the risk of overdose in such cases. Therefore, it is necessary to evaluate the effects of BCAA on the CNS with respect to HE treatment.

In this study, we aimed to shed light on the direct effects of BCAA on astrocyte survival and to identify the mechanism underlying the function of BCAA.

## 2. Methods

### 2.1. Cell Culture and Treatments

The human astrocyte cell line Cryo NHA (CLCC-2565) was purchased from Lonza Walkersville, Inc. (Walkersville, MD, USA). Cells were grown in a medium based on the AGM Bullet Kit (CC-3186) from the abovementioned company. The human hepatoblastoma cell line HepG2 was grown in DMEM supplemented with 10% fetal bovine serum (FBS, Thermo Fisher Scientific, New York, NY, USA). The mouse neuronal cell line HT22 was kindly provided by Dr. Takumi Satoh (Department of Welfare Engineering, Faculty of Engineering, Iwate University, Japan). Cells were grown in BCAA-free medium prior to BCAA treatment. Ammonium chloride (NH_4_Cl) was purchased from Sigma (St. Louis, MO), and BCAA (leucine-isoleucine-valine, 2 : 1 : 1.2) was kindly supplied by Ajinomoto Pharma Co. Ltd., Tokyo, Japan.

### 2.2. Evaluation of Viable Cells

Astrocytes, HepG2, and HT22 (1 × 10^4^ cells/sample) were treated with BCAA. Astrocytes were also treated with NH_4_Cl, BCAA, and a mixture of NH_4_Cl and BCAA. After 18 h of treatment, the number of viable astrocytes was evaluated using Cell Count Reagent SF (Nacalai Tesque Inc., Kyoto, Japan), as previously described [18]. 10 *μ*L of Cell Count Reagent SF was added to each well containing cultured astrocytes in 100 *μ*L medium. Cells were incubated for 1 h, and then, absorbance was measured at 450 nm using a microplate photometer (IMMUNO-MINI NJ-2300, Inter Med Co., Tokyo, Japan). Changes in cell number were arbitrarily represented using the optical density ratio, defined as the number of stimulated cells divided by that of control cells.

### 2.3. Lactate Dehydrogenase (LDH) Cytotoxicity Assay

Astrocytes (5 × 10^4^ cells/sample) were treated with NH_4_Cl, BCAA, and a mixture of NH_4_Cl and BCAA. After 4 h of treatment, LDH activity was detected using an LDH Cytotoxicity Assay Kit (Cayman Chemical, Ann Arbor, MI, USA). Absorbance was read at 490 nm using a microplate reader (IMMUNO-MINI NJ-2300, Inter Med Co., Tokyo, Japan).

### 2.4. Metabolome Analysis

Metabolomic screening was carried out through a facility service at Human Metabolome Technology (HMT) Inc., Tsuruoka, Japan. The experiment was carried out as described previously [12]. Astrocytes were treated with or without 0.2 mM BCAA. After 4 h of treatment, a dish of cultured cells (10^6^ cells/sample) was used for the extraction of intracellular metabolites. The culture medium was aspirated from the dish, and cells were washed twice with a 5% mannitol solution (10 mL first wash and 2 mL second wash). The cells were treated with 800 *μ*L of methanol and left at rest for 30 s to inactivate enzymes and extract the metabolites, according to the manufacturer's instructions. Next, the cell extracts were treated with 550 *μ*L of Milli-Q water containing internal standards (H3304-1002, Human Metabolome Technologies) and left at rest for another 30 s. The extract was centrifuged at 2,300 × *g* for 5 m at 4°C, and 800 *μ*L of the upper aqueous layer was centrifugally filtered through a Millipore 5 kD cutoff filter at 9,000 × *g* for 120 m at 4°C to remove proteins. The filtrate was concentrated through centrifugation and resuspended in 50 *μ*L of Milli-Q water for the Capillary Electrophoresis Time-of-Flight Mass Spectrometry (CE-TOFMS) (Agilent Technologies, Waldbronn, Germany) analysis. The detailed methods for CE-TOFMS have been described in previous publications [19, 20].

Peaks were extracted using the automatic integration software MasterHands (Keio University, Tsuruoka, Japan) to obtain peak information, including *m*/*z*, migration time for CE-TOFMS measurements (MT), and peak area. Signal peaks corresponding to adduct ions and other products of known metabolites were excluded, and the remaining peaks were annotated as putative metabolites and their isotopic ions from the HMT metabolic database based on their MT and *m*/*z* values determined by TOFMS. The tolerance range for the peak annotation was configured at ±0.5 m for MT and ±30 ppm for *m*/*z*. In addition, peak areas were normalized against those of the internal standards, and the resultant relative area values were further normalized to sample amounts.

### 2.5. Western Blot Analysis

Standard Western blotting was carried out as described previously [21]. Total protein was isolated from hepatocytes, using a total protein extraction kit (BioChain Institute, Newark, CA, USA) according to the manufacturer's instructions. Ten micrograms of protein from each sample was separated using 10% sodium dodecyl sulfate polyacrylamide gel electrophoresis (SDS-PAGE) and electrotransferred to a polyvinylidene difluoride membrane. Immunoblotting was performed using specific antibodies against Glut-1 (Abcam, Cambridge, UK), p-GSK-3*β*, and p-mTOR (Cell Signaling Technology Japan, K.K., Tokyo, Japan). The antibody against *β*-actin was purchased from Santa Cruz Biotechnology (Santa Cruz, CA, USA). Immunoreactive bands were visualized with an enhanced chemiluminescence reagent (GE Healthcare, Little Chalfont, Buckinghamshire, UK) and quantified using ImageJ software.

### 2.6. Statistical Analysis

Three technical replicates were set for each experiment (*n* = 3). All data are expressed as the mean ± standard deviation. Group comparisons were performed using Welch's *t*-test. Significance was defined as *p* < 0.05.

## 3. Results

### 3.1. BCAA Treatment Induces Cytotoxicity and Reduces Astrocyte Viability

**Figure 1:**
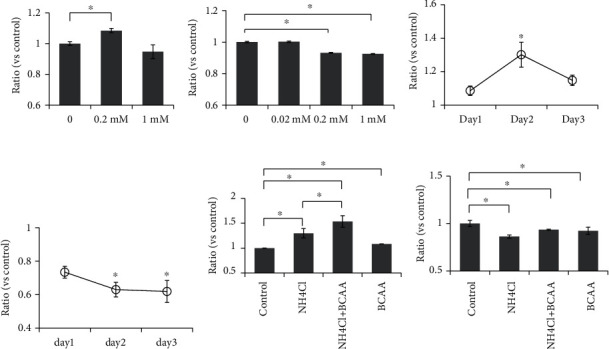
BCAA induce cytotoxicity and reduce astrocyte viability. LDH cytotoxicity assay (a) and evaluation of viable cells (b) among astrocytes following treatment with 0-1 mM BCAA. HT22 cells (c) and HepG2 cells (d) were treated with 0.2 mM BCAA for 24, 48, and 72 h prior to calculating cell viability. LDH cytotoxicity assay (e) and evaluation of viable cells (f) among astrocytes, following treatment with 0.2 mM BCAA, 10 mM NH_4_Cl, and BCAA+NH_4_Cl cotreatments. Control: no treatment with either BCAA or other reagents. Data are presented as the mean ± SD; *n* = 3; ^∗^*p* < 0.05.

Treatment of astrocytes with 0.2 mM BCAA induced a mean increase of 14% (*p* < 0.05) in LDH leakage (Figure 1(a)) and mean decrease of 9% (*p* < 0.05) in viability compared with that of nontreated (control) cells (Figure 1(b)). Concerning other cell types, 0.2 mM BCAA increased the proliferation of neuronal cells on the second day of treatment by approximately 30% (*p* < 0.05, Figure 1(c)), whereas it inhibited the proliferation of HepG2 cells (35% and 38% mean reductions were observed on days 2 and 3, respectively; *p* < 0.05; Figure 1(d)). Furthermore, BCAA and NH_4_Cl cotreatment resulted in enhanced cytotoxicity in astrocytes compared with both the control and NH_4_Cl treatment alone (*p* < 0.05, Figure 1(e)). Although BCAA slightly improved cell viability, which was reduced by ammonia, cell viability was still significantly lower than that in the control cells (*p* < 0.05, Figure 1(f)).

### 3.2. BCAA Treatment Affects Multiple Metabolic Pathways in Astrocytes

**Figure 2:**
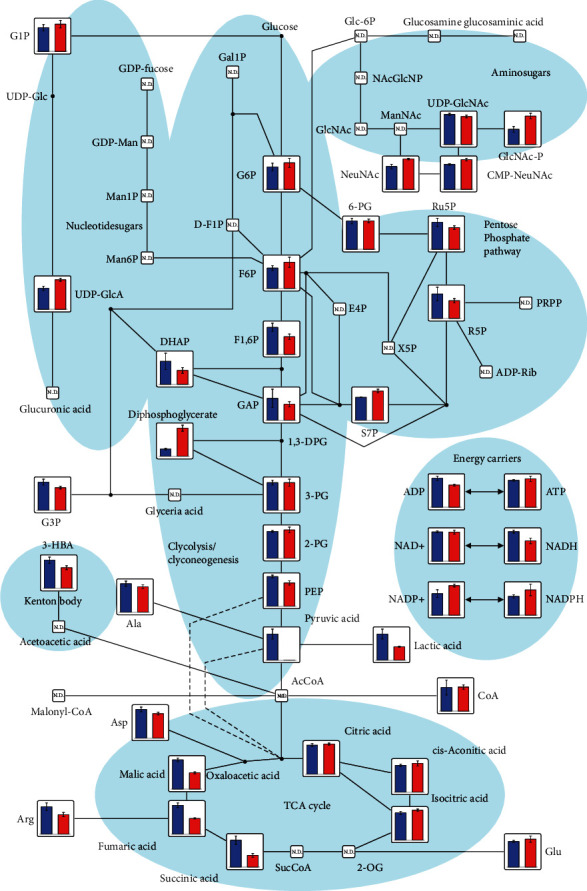
Map of central carbon metabolism pathways. Metabolites involved in the glycolysis/glyconeogenesis, TCA cycle, and central carbon metabolism are highlighted in the map. Blue bar: control (no treatment); Red bar: NH_4_Cl treatment. Data are presented as the mean ± SD. *n* = 3.

**Figure 3:**
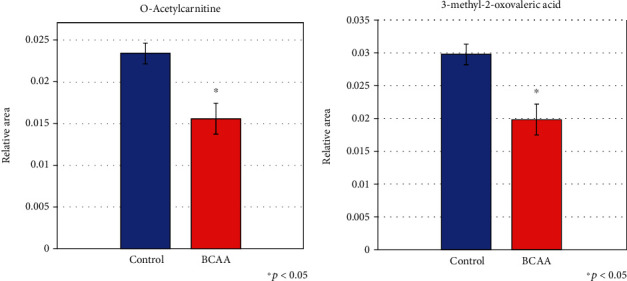
Effects of BCAA on the intracellular levels of neuroprotective and neural toxic metabolites. Intracellular levels of ALC (a) and 3-methyl-2-oxovaleric acid (b) were measured and compared with those in the control (no treatment) group. Data are presented as the mean ± SD. *n* = 3.

To identify the mechanism underlying the toxic effects of BCAA in astrocytes, we investigated metabolite expression levels in both control cells and in BCAA-treated astrocytes. We observed a significant disturbance in multiple metabolic pathways. First, we detected alterations in metabolites linked to central carbon metabolism. Despite the significant increase (*p* < 0.05) in the 2,3-diphosphoglyceric acid level, metabolites including fructose-1,6-diphosphate, phosphoenolpyruvic acid, and acetic acid, which are critical in glycolysis/glycogenesis mechanisms, were significantly decreased (*p* < 0.05) in BCAA-treated cells, compared to the control. As for energy carriers participating in ATP generation, which is the major function of mitochondria, despite ATP and NAD^+^, which showed no obvious difference between the BCAA-treated and nontreated cells, all other components including ADP, fumaric acid, NADH, and succinic acid were significantly decreased (*p* < 0.05) upon BCAA treatment. In addition, metabolites of the tricarboxylic acid (TCA) cycle, such as malic acid, fumaric acid, and succinic acid, showed a significant decrease in BCAA-treated cells (Figure 2). Second, regarding intracellular protein metabolism, the relative levels of almost all intracellular amino acids, including BCAA, were decreased in BCAA-treated cells compared with the control (Table 1). Third, in terms of intracellular lipid metabolism, although levels of metabolites related to fatty acid synthesis, such as decanoic acid and lauric acid, were not significantly changed after BCAA treatment (data not shown), metabolites of the butyric (short-chain fatty acids) pathway, such as GABA, showed lower levels in BCAA-treated cells than in the control (Table 2). Additionally, the level of carnitine-derivative acetylcarnitine (ALC), which exerts neuroprotective effects by inducing ureagenesis and improving energy metabolism [22, 23], was decreased in BCAA-treated cells. Notably, 3-methyl-2-oxovaleric acid, which is mainly produced from isoleucine and plays a critical role in neurological damage [24], was also decreased upon BCAA treatment (Figure 3).

### 3.3. Effects of BCAA Treatment on GSK-3/mTOR Signaling in Glut-1 Regulation

**Figure 4:**
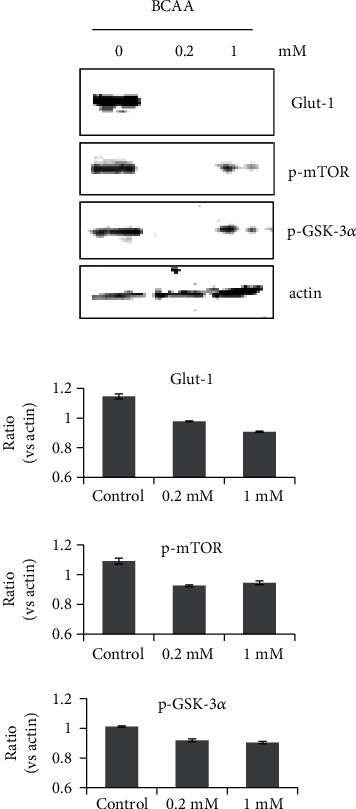
Effects of BCAA on the protein levels of Glut-1 and activation of GSK-3*β* and mTOR. Western blot analysis of Glut-1 (60 kDa), phosphorylated GSK-3*β* (46 kDa), and mTOR (289 kDa) from extracted astrocytes, following 24 h treatment with 0.2 mM BCAA. *β*-Actin (43 kDa) was used as a loading control. The blots were cropped after stripping (Re-blot plus mild solution, Thermo Fisher Scientific Inc., Goteborg, Sweden) and restaining the same membrane. The protein bands from Western blot films were quantified with ImageJ (NIH, USA). Control: no treatment. *n* = 3.

Glucose uptake, uniquely regulated by Glut-1, is an important function of astrocytes [25]. As our metabolomic analysis showed a disturbance in glycolysis/glyconeogenesis, we further investigated the possible effects of BCAA on Glut-1 production. The results of this experiment showed that an 18 h treatment with BCAA decreased Glut-1 protein levels. Moreover, signaling molecules linked to Glut-1 production and regulation, such as GSK-3*α*/*β* and mTOR [26], were hypophosphorylated compared to the control (Figure 4).

## 4. Discussion

BCAA supplementation, especially BCAA infusion therapy, has been reported to improve the symptoms of HE induced by hyperammonemia [4–6]. However, the mechanisms underlying the direct effect of BCAA on the CNS have rarely been investigated. The vast majority of studies on ammonia toxicity in HE studies have focused on the effects of ammonia toxicity on astrocytes [27, 28]. We have previously shown that ammonia increases cytotoxicity in human astrocytes and inhibits their proliferation [12]. In this study, we had initially predicted that BCAA would exert a protective effect on the astrocytes especially against hyperammonemia; however, our data strongly suggests that BCAA induces cytotoxicity, impairs astrocyte survival, and enhances the cytotoxic effect of NH_4_Cl on astrocytes. BCAA treatment slightly, but did not significantly, improved the antiproliferative effects of NH_4_Cl in astrocytes, as shown in Figure 1(f); although we could not identify the reason for this, it was clear that the effects of BCAA were too weak to aid total recovery of the level of proliferation of the astrocytes to that of the control.

On the other hand, BCAA has been reported to prevent hepatocarcinogenesis and prolong the survival of patients with cirrhosis [29]. Similarly, our data showed that BCAA inhibited the proliferation of human hepatoblastoma cells. Our *in vitro* data using two cell lines indicate that the anticancer effects of BCAA may be accompanied by side effects of neural damage. In addition, the negative impact of high BCAA levels on neurons is supported by recent findings based on cultured neurons and motor cortex slices from mice fed a BCAA-enriched diet [30]. Conversely, the present study showed a positive effect on the proliferation of mouse neuronal cells. Therefore, further studies are needed to confirm and clarify the effects of BCAA on both mouse and human neurons.

Metabolome analysis using blood and tissue samples has recently been developed, allowing to comprehensively evaluate the pathways of carbohydrate, AA, nucleotide, and lipid metabolism [12, 31]. However, no reports have described the use of this method to measure the effects of BCAA on intracellular metabolites in astrocytes. Our previous study revealed that ammonia induces an abnormal intracellular increase in BCAA and its neural toxic derivatives, such as 3-methyl-2-oxovaleric acid [12]. Nonetheless, both BCAA and 3-methyl-2-oxovaleric acid were herein found to decrease with BCAA treatment. Our data therefore indicate that BCAA negatively affects astrocyte survival potentially by perturbing the cell metabolism, but not by toxic compound accumulation in astrocytes. In the present study, we elucidated the effects of BCAA on intracellular metabolism in this study with a single dose, single time point, and the limited repeated samples (*n* = 3) because of the high cost of the metabolism assay. Further studies should be conducted to completely understand the effects of BCAA on intracellular metabolism.

Astrocytes play a central role in controlling brain energy metabolism; this is supported by *in vivo* observations indicating that astrocytes are primarily responsible for glucose uptake in the brain [32] and that astrocytes take up half of the glucose from capillaries. Astrocytes are distinguished from neurons in the CNS based on their glucose uptake from the circulation through the Glut-1 glucose transporter. It has also been reported that Glut-1 silencing inhibits cell proliferation and glucose uptake [33]. In this study, Glut-1 protein levels in astrocytes decreased upon BCAA treatment. This indicates that the glucose uptake in astrocytes was impaired, which may be related to the disturbance of glycolysis/glycogenesis. The mTOR/GSK-3 pathway reportedly downregulates Glut-1 synthesis. However, upon BCAA treatment, the total protein levels of Glut-1 and the phosphorylation levels of mTOR and GSK-3 were all decreased. Our data suggests that the mTOR/GSK-3 pathway may not function upstream of Glut-1 in astrocytes upon BCAA treatment. Therefore, the mechanism of how BCAA affects glucose uptake and the correlation with the mTOR/GSK-3 signaling pathway should be further investigated.

In addition, we found evidence of decreased ALC levels upon BCAA treatment, which suggests a disturbance in intracellular carnitine metabolism. Carnitine deficiency has been proven to result in HE [34]; ALC plays an important role in fat metabolism and mitochondrial energy production [22] and has been considered a therapy for patients with overt HE or covert HE as it induces ureagenesis and improves energy metabolism [35]. We recently reported that carnitine reduced cytotoxicity and intracellular metabolism impairment caused by ammonia. We also found that carnitine treatment significantly increased the protein levels of Glut-1 in astrocytes (data not shown). Our data indicates the possibility that carnitine could prohibit the negative effects of BCAA on astrocytes.

In conclusion, although BCAA supplementation for HE treatment caused by LC and/or portal-systemic shunt has benefits that improve consciousness levels [36], the present study shows that BCAA has direct negative effects on astrocyte survival and intracellular metabolism, which suggests a limitation in HE. A further study to clarify the effects of BCAA on human astrocytes with hyperammonemia is needed, because BCAA supplementation showed no prevention for ammonia-induced toxicity in astrocytes.

## Figures and Tables

**Table 1:** Relative changes of intracellular amino acids in astrocytes.

Name of amino acids	BCAA vs. control ratio
Lysine	0.7
Arginine	0.7^∗^
Histidine	0.8
Aspartate	0.9
Glutamate	1.1
Serine	0.9
Threonine	0.9
Asparagine	0.9
Glutamine	0.9
Alanine	0.9
Glycine	0.9
Valine	0.8
Isoleucine	0.8
Leucine	0.8
Phenylalanine	0.7
Tyrosine	0.7^∗^
Tryptophan	0.8^∗^
Methionine	0.8
Cysteine	1.4
Proline	0.8

Data expressed as the mean value (*n* = 3). ^∗^*p* < 0.05 (Welch's *t*-test).

**Table 2:** Levels of metabolites related to butyric acid metabolism.

Compound name	Comparative analysis		
	BCAA vs. control		
	Ratio	*p*value	
2-Hydroxyglutaric acid	1.0	0.884	
3-Hydroxybutyric acid	0.7	0.031	^∗^
Fumaric acid	0.5	0.008	^∗∗^
GABA	0.9	0.158	
Glu	1.1	0.298	
Malic acid	0.6	0.001	^∗∗^
Pyruvic acid	<1	N.A.	
Succinic acid	0.4	0.014	^∗^

Data expressed as the mean value (*n* = 3). ^∗^*p* < 0.05 and ^∗∗^*p* < 0.01 (Welch's *t*-test).

## Data Availability

The data used to support the findings of this study are included within the article.
